# Obituary, Etc.

**Published:** 1870-11

**Authors:** 


### *Obituary.*

Charles H. Ray, M.D., Editor-in-chief of the Chicago *Evening Post*, died in this city, Sept. 23. Dr. Ray was born in Norwich, Chenango Co., N. Y., March 12, 1821, and removed to the West in 1843, commencing the practice of medicine at Muscatine, Iowa, but subsequently settling in Tazewell Co., Ill., where he pursued the practice of his profession successfully for several years. In 1851, he removed to Galena, and, somewhat accidentally, was induced to enter the editorial profession. In 1855 he became connected with the *Chicago Tribune*, with which he remained identified until Nov., 1863, when he sought other pursuits, but these not proving pecuniarily successful, in 1867 he returned to the editorial profession as Editor-in-chief of the *Evening Post*, which position he occupied at the time of his death. In January last, he was prostrated by "brain fever," but after some months he recovered so far as to resume his editorial duties. The immediate sickness which resulted in his death, commenced on Tuesday with fatal termination the ensuing Friday. *Post mortem* examination showed, however, that the previous attack had left permanent lesions, which, if known, would have precluded hope of recovery, although total cessation of mental labor might have prolonged his life. But it is difficult to imagine a brain, as active as was his, ever abstaining from work.

Had Dr. Ray continued in the practice of his profession, and employed, as he would have done, his extraordinary powers of mind in its scientific development, he would have easily grasped the highest honors it has to offer. Always, however, he was alive to every advance, and cheered the steps of progress. A writer in the *Tribune*, with which he was so long connected, and who knew him well, so adequately expresses our own ideas that we are glad to use the language which he furnishes to our hand:

"In our professional association with him, which has extended over many years, we learned to prize him as a man, and to hold him dear as a friend. He was not one, perhaps, to attract numerous friendships, for he was brusque and impetuous in his manner, and specially impatient of annoyance. But those who knew him best, knew how genial he was at heart, how strong his affections were, and how almost faultless he was in critical taste. He was intense in his likes and dislikes. He was bitter against an enemy, but he could not do too much for a friend. We have seen him fairly crush insincerity with an explosion of his wrath, and then turn and relieve the wants of a traveling beggar, and give him kindly advice. He was the best friend a young man commencing newspaper life could have, for the reason that he was chary of praise and never slow at pointing out faults and suggesting the remedy. Perhaps the most striking feature of his character was his hatred of cant and sham. He recognized a hypocrite instinctively, and he never stopped to select choice or elegant phrases in exposing him. We cannot remember a man so plain-spoken in denunciation of humbug or hypocrisy. He hit it with all his might, and his might was immense. And yet, this Samson was full of humanity, kindly courtesy, and noble, hearty manliness. With all his multifarious duties, private and public, which were often very perplexing, he found time to devote much attention to literature and art, and in these directions his taste was fastidious, and his manner quick and resolved. He was as impatient of sham in a book, in a painting, or in the music room, as he was of sham in life, and his criticism was almost always just, even though it was excoriating. The class of men who cannot be politic enough to compromise with hypocrisy is so scarce that it is refreshing to recall this trait in Dr. Ray's character. It made enemies, of course, but that was of little account to him. The man who has no enemies must be all things to all men. He was a hard worker, and, in his prime, was capable of an immense amount of labor, for he was physically very strong. Few men in the journalistic profession, indeed, have combined such power to labor, such keen perceptions, such a nervous, trenchant style, and such manly and vigorous grappling with private and public evils.

Just such men as Dr. Ray was, are needed in the medical profession, and we would hold up the traits of character which characterized and ennobled our deceased friend to the emulation of all young men who link their future with its destinies.

### *New York State Inebriate Asylum.*

The following announcement is respectfully submitted to the consideration of the medical profession, and to the public in general:

It is believed that the experience of the past five years has demonstrated not only the utility but the necessity of the institution known as the New York State Inebriate Asylum. We speak advisedly when we affirm that at no time has its prospects for usefulness been more promising, or has it been in so good a condition, so far as the treatment of patients is concerned, as it is now. We have sought to make it what it was originally intended to be, a reformatory christian home.

There are very many persons in our State and throughout the country, the victims of a terrible mania for drink, who need the salutary treatment which this Institution affords, and who, without such aid, must in all human probability perish. We, therefore, disclaiming every object except an earnest desire to aid in restoring to their friends and to society a class of men fallen indeed, but not beyond recovery, would earnestly commend this Institution as an efficient means for securing an end so important and inestimable.

We deem it proper to state, that ample means are provided to meet the physical, intellectual and religious wants of the patients. The Asylum occupies a remarkably healthful and beautiful site. It is furnished with baths, and a great variety of amusements; with a good library and reading room, which is supplied with the leading daily newspapers and the American and British magazines.

The rules of the Institution require *regularity* in regard to meals—the hours of retiring and rising—and the attendance on the religious exercises of the establishment.

The Asylum has been placed under the charge of Dr. Daniel G. Dodge, a man of superior administrative qualifications, and toward whom there is but one sentiment prevailing with the officers of the Institution and among the patients, that of profound respect for him as a christian gentleman, and confidence in him as a skillful physician.

Binghampton, N. Y., Oct. 1, 1870. WILLARD PARKER, M.D.,
New York, N. Y.,
REV. SAMUEL W. BUSH, *Register.* *President Board Trustees.*

### *To Soldiers and Sailors of Illinois who lost limbs in the late war.*

At a meeting of the Illinois State Republican Association, held September 6, 1870, in Liberty Hall, Washington, D. C., the following resolutions were adopted:

*Resolved*, That a committee of three be appointed, of which the Corresponding Secretary shall be a member, to draft a circular letter, and forward it to the different newspapers in Illinois for publication, tendering to every soldier or sailor in the State who has lost a leg or an arm, the assistance of this association to enable them to take advantage, free of all expense to themselves, of the late act of Congress giving them artificial limbs, or a commutation in money instead.

*Resolved*, That this committee be constituted a permanent committee, and it shall be their duty to report to the association from time to time upon their action.

The President appointed, as said committee, A. B. Wicker, J. M. McNeill, Junius Simons.

The Committee, in carrying out the objects of the resolutions, would say to all those for whose benefit the action was taken, that Congress, in the exercise of its beneficence toward that class of our fellow-citizens, intended that they should have the benefit of the act free of any expense, and so framed the law that the intervention of attorneys and claim agents would not be necessary to the adjustment of the claims.

Applications should be made by officers of the Army and soldiers direct to the Surgeon-General, from whose office the necessary blanks will be furnished on request. Officers of the Navy and seamen should apply for blanks to the Chief of the Bureau of Medicine and Surgery, Navy Department, Washington.

Any further information desired or assistance wanted in the procurement of the artificial limbs or commutation in money, provided by the act, can be had by addressing any one of the committee at Washington, D. C.

